# Supplementary material for: Contacts-based prediction of binding affinity in protein–protein complexes
Source: eLife. 2015 Jul 20;4:e07454. doi: 10.7554/eLife.07454 (PMC4523921; doi:10.7554/eLife.07454)
Supplement: Supplementary file 1. — List of entries removed from the original binding affinity benchmark (Alberts, 1998) because of gaps at interface. DOI: http://dx.doi.org/10.7554/eLife.07454.012 [file elife-07454-supp1.docx]

**Supplementary file 1**

List of entries removed from the original binding affinity benchmark^1^ because of gaps at interface.

| **PDB code** | **Typology** | **Chain** | **Gap residues** |
| --- | --- | --- | --- |
| 1F34 | ligand | B | 123-132 |
| 1HCF | receptor | A | 65-70 |
| 1H9D | ligand | B | 71-79 |
| 1KLU | ligand | D | 97-105 |
| 1NVU_QS | ligand | S | 744-749 |
| 1NVU_RS | ligand | S | 591-596 |
| 1NW9 | receptor | B | 289-315+321-332 |
| 1R6Q | ligand | C | 14-19 |
| 1T6B | receptor | X | 276-288 |
| 1WDW | ligand | A | 167-172 |
| 2HLE | ligand | B | 96-99 |
| 2I9B | receptor | E | 132-136+106-109 |
| 2OZA | receptor/ligand | B/A | 173-180/270-277 |
| 1JMO | receptor | A | 1-54 |
| 1RV6 | ligand | X | 225-229 |
| 2B4J | receptor | A | 187-194 |
| 3BP8 | receptor | A | 65-75 |
| 1KXP | receptor | A | 365-375 |
| 2B42 | receptor | A | 263-265 |

1. Kastritis, P. L. *et al.* A structure-based benchmark for protein-protein binding affinity. *Protein Sci* **20**, 482-491, doi:10.1002/pro.580 (2011).
