# Supplementary material for: Contacts-based prediction of binding affinity in protein–protein complexes
Source: eLife. 2015 Jul 20;4:e07454. doi: 10.7554/eLife.07454 (PMC4523921; doi:10.7554/eLife.07454)
Supplement: Supplementary file 3. — Table summarizing the weights (wN) and performance (expressed as Pearson's coefficient R and RMSE) of the fourfold cross-validation, repeated 10 times, of the following binding affinity regression model: ΔGcalc=w1ICscharged/charged+w2 ICscharged_apolar−w3 ICspolar/polar+w4 ICspolar/apolar+w5 %NISapolar+w6 %NIScharged+Q.Each coefficient has been reported as average on the four models trained on the respective folds. DOI: http://dx.doi.org/10.7554/eLife.07454.014 [file elife-07454-supp3.docx]

**Supplementary file 3**

Table summarizing the weights (w_N_) and performance (expressed as Pearson’s Coefficient R) of the 4-fold cross-validation, repeated 10 times, of the following binding affinity regression model:

ΔG_calc_ = w_1_ ICs_charged/charged_ + w_2_ ICs_charged_apolar_ – w_3_ ICs_polar/polar_ + w_4_ ICs_polar/apolar_

+ w_5_ %NIS_apolar_ + w_6_ %NIS_charged_ + Q

Each coefficient has been reported as average on the four models trained on the respective folds.

|  | ***W_1_*** | ***W_2_*** | ***W_3_*** | ***W_4_*** | ***W_5_*** | ***W_6_*** | R  _training_ | R  _prediction_ |
| --- | --- | --- | --- | --- | --- | --- | --- | --- |
| **4-fold1** | 0,09501 | 0,09982 | -0,19803 | 0,22253 | -0,19601 | -0,14249 | 0,75 | 0,68 |
| **4-fold2** | 0,09737 | 0,09845 | -0,18927 | 0,22174 | -0,18422 | -0,14123 | 0,74 | 0,68 |
| **4-fold3** | 0,09543 | 0,09781 | -0,20434 | 0,22816 | -0,18306 | -0,13461 | 0,74 | 0,70 |
| **4-fold4** | 0,09153 | 0,10596 | -0,18779 | 0,21853 | -0,18745 | -0,14614 | 0,75 | 0,66 |
| **4-fold5** | 0,09224 | 0,10091 | -0,19019 | 0,21989 | -0,18913 | -0,13950 | 0,74 | 0,65 |
| **4-fold6** | 0,09502 | 0,10067 | -0,19308 | 0,22218 | -0,19375 | -0,13557 | 0,75 | 0,68 |
| **4-fold7** | 0,09929 | 0,10402 | -0,17989 | 0,21283 | -0,18783 | -0,14139 | 0,75 | 0,65 |
| **4-fold8** | 0,07066 | 0,09868 | -0,19148 | 0,22295 | -0,18923 | -0,13625 | 0,74 | 0,71 |
| **4-fold9** | 0,09859 | 0,09860 | -0,18712 | 0,22188 | -0,18312 | -0,14454 | 0,74 | 0,70 |
| **4-fold10** | 0,09171 | 0,09868 | -0,19324 | 0,22670 | -0,18795 | -0,13899 | 0,75 | 0,65 |
|  |  |  |  |  |  |  |  |  |
| **Average** | 0,09268 | 0,10036 | -0,19144 | 0,22174 | -0,18817 | -0,14007 | 0,74 | 0,67 |
| **Standard**  **deviation** | 0,008 | 0,003 | 0,007 | 0,004 | 0,004 | 0,004 | 0,00 | 0,02 |
