# Supplementary material for: Contacts-based prediction of binding affinity in protein–protein complexes
Source: eLife. 2015 Jul 20;4:e07454. doi: 10.7554/eLife.07454 (PMC4523921; doi:10.7554/eLife.07454)
Supplement: Supplementary file 5. — List of Fab D3H44 antibody residues in the binding interface of the complex with the Tissue factor (PDB code of the complex: 1JPS). For each residue N, its relative contribution (expressed as a percentage) to the total number of inter-residue contacts made and to the total buried surface area of the Fab is reported. ICN and BSAN are the interface contacts and the buried surface area of residue N, respectively; ICtotal is 83; BSAFab_total is evaluated as half of the BSA for the complex corresponding to 926 Å2. DOI: http://dx.doi.org/10.7554/eLife.07454.016 [file elife-07454-supp5.docx]

**Supplementary file 5**

List of Fab D3H44 antibody residues in the binding interface of the complex with the Tissue factor (PDB code of the complex: 1JPS). For each residue N, its relative contribution (expressed as a percentage) to the total number of inter-residue contacts made and to the total buried surface area of the Fab is reported. IC_N_ and BSA_N_ are the interface contacts and the buried surface area of residue N, respectively; IC_total_ is 83; BSA_Fab_total_ is evaluated as half of the BSA for the complex corresponding to 926 Å^2^.

| Residue type | | %ContributionBSA | | | %Contribution  ICs |
| --- | --- | --- | --- | --- | --- |
| ARG | L27 | | 1,1 | 1,2 | |
| LYS | L30 | | 2,5 | 2,4 | |
| TYR | L32 | | 7,7 | 4,8 | |
| TYR | L50 | | 3,0 | 1,2 | |
| HIS | L91 | | 1,1 | 1,2 | |
| GLY | L92 | | 3,3 | 3,6 | |
| GLU | L93 | | 2,4 | 3,6 | |
| SER | L94 | | 4,3 | 4,8 | |
| TRP | L96 | | 1,6 | 2,4 | |
| PHE | H27 | | 0,1 | - | |
| ILE | H29 | | - | 1,2 | |
| LYS | H30 | | 0,8 | 1,2 | |
| GLU | H31 | | 10,5 | 6,0 | |
| TYR | H32 | | 5,6 | 6,0 | |
| TYR | H33 | | 8,1 | 6,0 | |
| LEU | H50 | | 1,6 | 2,4 | |
| ASP | H52 | | 2,2 | 4,8 | |
| PRO | H53 | | - | 1,2 | |
| GLU | H54 | | 6,5 | 6,0 | |
| GLN | H55 | | 3,1 | 3,6 | |
| ASN | H57 | | 7,3 | 6,0 | |
| THR | H58 | | 1,7 | 2,4 | |
| ILE | H59 | | 7,0 | 4,8 | |
| TYR | H60 | | 0,2 | 1,2 | |
| PRO | H62 | | 0,5 | - | |
| GLN | H65 | | 3,5 | 3,6 | |
| ARG | H98 | | 0,5 | - | |
| ASP | H99 | | 0,1 | 2,4 | |
| THR | H100 | | 4,2 | 4,8 | |
| ALA | H101 | | 6,5 | 7,2 | |
| ALA | H102 | | 2,0 | 3,6 | |
